# Unsuccessful tracheal intubation in a patient with Kniest dysplasia undergoing repeated general anesthesia: a case report

**DOI:** 10.1186/s40981-018-0178-x

**Published:** 2018-05-18

**Authors:** Maiko Hasegawa-Moriyama, Tomonori Iwasaki, Keika Mukaihara, Mina Masuda, Yuichi Kanmura

**Affiliations:** 1 Department of Anesthesiology and Critical Care Medicine, Graduate School of Medical and Dental Sciences, Kagoshima University, 8-35-1 Sakuragaoka, Kagoshima, 890-8520 Japan; 2 Department of Pediatric Dentistry, Graduate School of Medical and Dental Sciences, Kagoshima University, 8-35-1 Sakuragaoka, Kagoshima, 890-8520 Japan

**Keywords:** Kniest dysplasia, Collagenopathy, Difficult airway

## Abstract

**Background:**

Kniest dysplasia is a type of chondrodysplasia characterized by severe craniofacial abnormalities including tracheomalacia, midface hypoplasia, and cleft palate.

**Case presentation:**

We previously described a 6-year-old girl with Kniest dysplasia, in whom glottic edema rapidly developed after tracheal intubation. At the age of 13 years, a reoperation was scheduled to correct talipes equinovarus but was subsequently canceled due to failure of tracheal intubation and subsequent glottic edema. Airway evaluation by endoscopy and computed tomography 1 month later revealed severe laryngeal narrowing. Therefore, the second anesthesia was maintained with spinal anesthesia combined with sciatic nerve block without tracheal intubation.

**Conclusion:**

Careful perioperative airway evaluation is required in patients with Kniest dysplasia, and alternative strategies for airway management other than tracheal intubation should be considered.

## Background

Kniest dysplasia is a form of chondrodysplasia caused by a type II collagenopathy that develops in patients with a COL2A1 gene mutation [1]. Kniest dysplasia is characterized by kyphoscoliosis, a short trunk and limbs, severe craniofacial abnormalities, myopia, and hearing impairment [2, 3]. The craniofacial and cervical abnormalities include tracheomalacia, midface hypoplasia, and cleft palate. Atlantoaxial instability is also associated with this syndrome [4]. We previously described a 6-year-old girl with Kniest dysplasia who developed laryngeal edema after tracheal intubation for gastrocnemius myotomy to correct talipes equinovarus and required postoperative airway management in the intensive care unit [5]. We herein report our experience with the same patient at the age of 13 years. Tracheal intubation repeatedly failed due to narrowing of the glottis, resulting in glottic edema and a 1-month delay in performing surgery.

## Case presentation

A 13-year-old girl (height, 151 cm; weight, 40.3 kg) with Kniest dysplasia was scheduled for surgery to recorrect talipes equinovarus. A flat nasal root and mandibular prognathism were present, but no symptoms of airway obstruction such as stridor, wheezing, or respiratory distress were detected preoperatively. The patients had no associated cartilage abnormalities, such as cleft palate, laryngomalacia, tracheomalacia, micrognathia, or platyspondyly.

**Fig. 1:**
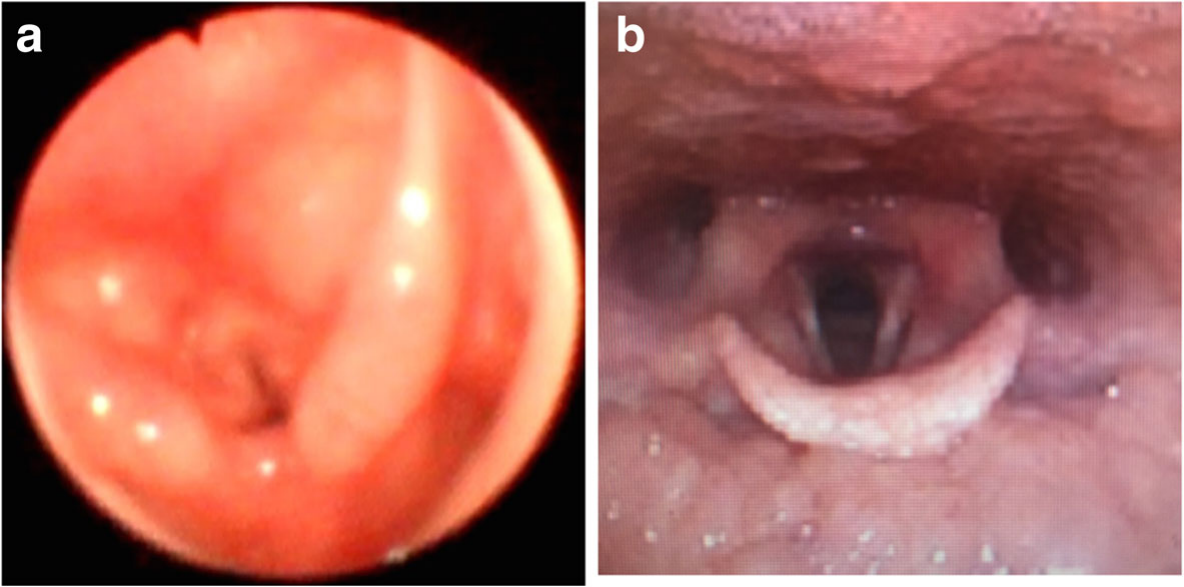
The larynx of the patient with Kniest dysplasia was endoscopically evaluated immediately after two trials of tracheal intubation **a** in the operating room and **b** in the outpatient room 1 month later

She had previously undergone the same surgery at the age of 6 years, at which time laryngeal edema had rapidly developed after tracheal intubation, resulting in intensive care unit admission with tracheal intubation for 21 days. On the day of the scheduled reoperation at 13 years of age, her oxygen saturation upon entering the operating room was 99% on room air. Anesthesia was induced by intravenous administration of remifentanil at 0.5 μg/kg/min, thiopental at 200 mg, and rocuronium at 30 mg. Mask ventilation and laryngoscopy were easily performed. However, despite two attempts to perform tracheal intubation using uncuffed 6.0 and 6.5 mm standard tracheal tubes, tracheal intubation could not be achieved because of narrowing of the glottis. The tip of the tracheal tube did not pass the glottic level. False vocal cord swelling began after only two attempts and considering the history of prolonged laryngeal edema that had developed at the age of 6 years in this patient, the trial of tracheal intubation was discontinued. After placement of a size 2.5 laryngeal mask, anesthesia was maintained with 2.5% sevoflurane, and the airway was evaluated by a fiberscope 2 h later under spontaneous breathing through the laryngeal mask before the end of anesthesia. Although the end-tidal CO_2_ level was maintained at 40 to 46 mmHg and the capnogram waveform was plateaued without exhibiting an obstructive pattern or increase in the peak airway pressure, the development of laryngeal edema and glottic narrowing was observed using a fiberscope (Fig. 1a). Hydrocortisone 150 mg was intravenously administered, and the laryngeal mask was removed 139 min after the trials of tracheal intubation. We confirmed that the patient’s respiratory condition was not disturbed and returned her to the general ward. Her oxygen saturation upon exiting the operating room was 99% with the oxygen mask at 3 L/min. No postoperative exacerbation of her respiratory condition or decline in her oxygen saturation level was observed. Surgery was delayed until 1 month later.

**Fig. 2:**
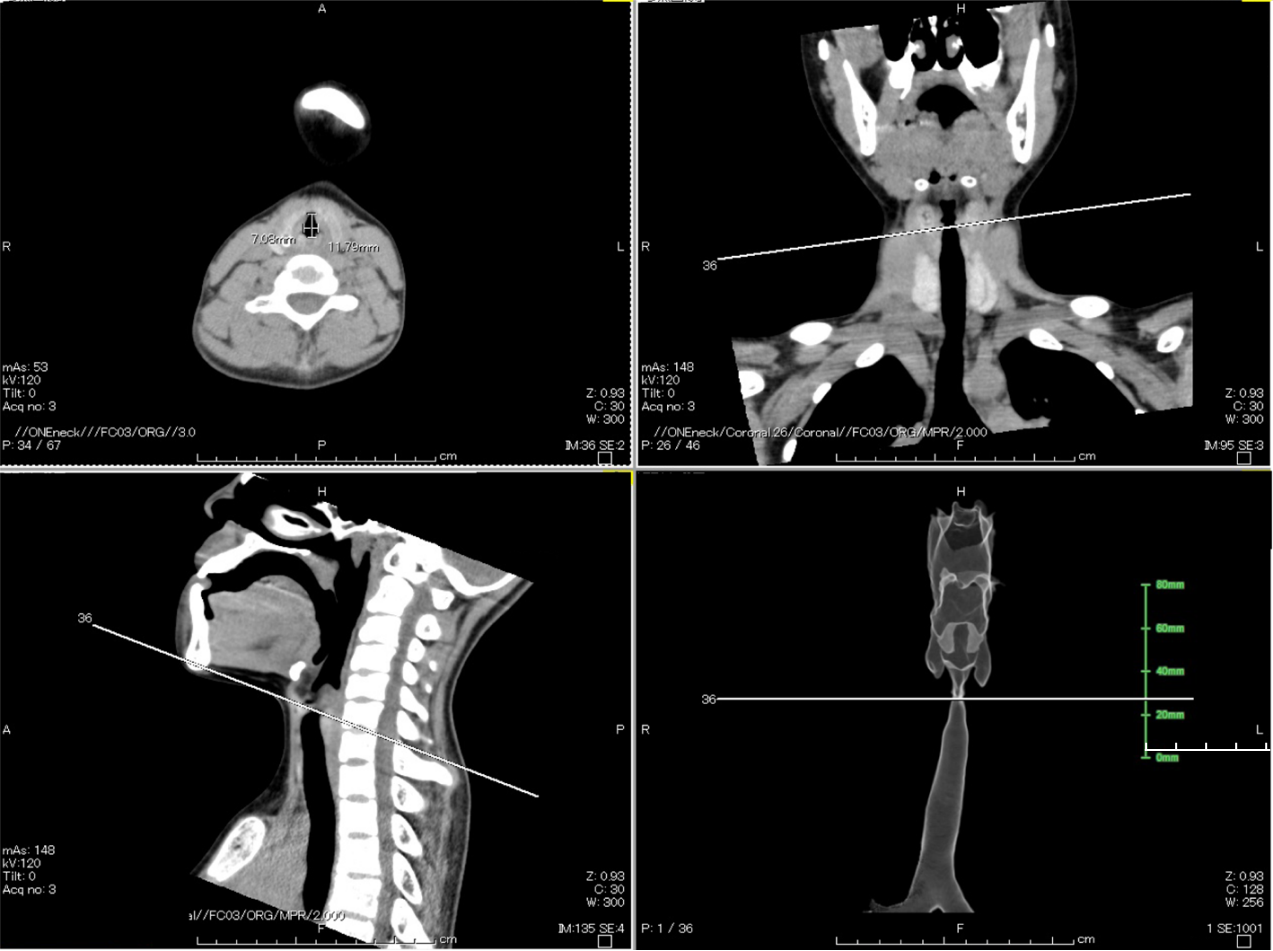
Cervical computed tomography showed the narrowest site of the airway was in laryngeal region, not at the lower airway levels

1 month later, nasal endoscopy was performed by an otolaryngologist the day before the scheduled surgery. The otolaryngologist pointed out the small larynx and narrow glottis, but no vocal cord edema, thickness, or immobilization was observed throughout the laryngopharynx (Fig. 1b). In addition, no airway narrowing or deformity was detected from the glottis to the carina by cervical computed tomography (Fig. 2). The airway was not compressed by the curve of the cervical vertebrae, and cervical retroflexion was not impaired preoperatively. The minimum diameter of the airway on the axial view was 7.08 mm (anteroposterior diameter) and 11.79 mm (transverse diameter) as identified at the glottic level (Fig. 2). The second anesthesia was performed without tracheal intubation. Anesthesia was induced with midazolam 2 mg, fentanyl 25 mg, and dexmedetomidine 2 μg/kg/h, and spinal anesthesia was then performed at the L3/4 level with 2.5 ml of 0.5% bupivacaine. The catheter used for the sciatic nerve block was inserted by a subgluteal approach for postoperative analgesia. The patient was sedated with dexmedetomidine at 0.5 to 1.2 μg/kg/h during surgery. Adequate spontaneous breathing was maintained without symptoms of laryngospasm or airway obstruction such as stridor or wheezing and the oxygen saturation was maintained at > 99% during anesthesia. The surgery time was 175 min, and the anesthesia time was 209 min. The patient was transferred to the general ward for postoperative care without airway complications.

## Discussion

Perioperative airway assessment is particularly important in patients with congenital anomalies that may cause obstruction. Although collagenopathies are reportedly associated with impaired musculoskeletal development such as kyphoscoliosis, tracheomalacia, midface hypoplasia, and cleft palate [6], which are likely to induce difficult airway management, rapid development of airway edema during airway management has rarely been described. We previously reported the development of airway constriction after several trials of tracheal intubation in the present patient associated at the age of 6 years [4]. At that time, it was not clear whether the laryngeal and epiglottic edema was due to soft tissue vulnerability caused by the collagenopathy or by the repeated trials of tracheal intubation, which can develop independent of Kniest dysplasia.

We experienced failure of tracheal intubation and glottis edema again after only two trials of tracheal intubation when this patient was 13 years of age. The Japanese Society for Anesthesiologists (JSA) airway management guideline recommends preoperative airway evaluation by a modified Kheterpal’s model to predict combined difficult facemask ventilation and direct laryngoscopy [7]. In our patient, none of the 12 assessment factors of this model were found, and facemask ventilation and direct laryngoscopy were easily performed. However, endoscopic examination revealed that the larynx was small. These episodes suggest that immature development of the airway can be preoperatively diagnosed even at a higher age and that perioperative rapid development of airway edema should be expected regardless of age in patients with Kniest dysplasia. Airway management with tracheal intubation rather than a laryngeal mask was chosen at the age of 13 years because preoperative evaluation by physical examination did not indicate airway constriction. However, a supraglottic airway device should have been used without attempting tracheal intubation regardless of the patient’s age because of the soft tissue vulnerability caused by the collagenopathy. In addition, the 2014 JSA airway management guideline 2014 states that the capnogram waveform provides only limited information in the neonatal and pediatric population and that the ventilation status grades in these groups of patients should therefore be determined together with any other clinical information available. Although the capnogram wave form was maintained at a plateau without exhibiting an obstructive pattern or increase in peak airway pressure, the possible development of airway edema should be considered in patients with a collagenopathy.

Segawa et al. [8]. reported a case involving a patient with Kniest dysplasia in which increased difficulty in performing tracheal intubation occurred as the patient aged. Although tracheal intubation was easily performed at the age of 6 years, only the tip of the epiglottis was observed with the laryngoscope, and the trachea was blindly intubated at the age of 11 years. This implies that anatomical changes of the larynx with growth may cause difficult airway management in patients with Kniest dysplasia. Whether mucosal irritation by repeated tracheal intubation at a younger age led to the laryngeal thickening and airway narrowing is unclear, but the small larynx noted by the otolaryngologist suggests that impaired laryngeal growth was present in this patient. The edema around the vocal cords persisted for 9 days, and the tracheal tube was extubated on postoperative day 21 at the age of 6 years. The prolonged contact of the tracheal tube with the mucosa at the level of the glottis may have exacerbated the edematous changes that were induced by tracheal intubation. Therefore, even if organic constriction is not detected by preoperative evaluation, tracheal intubation should be avoided as much as possible.

Patients with Kniest dysplasia often require several operations because of multiple osteochondral tissue anomalies. Type II collagen also plays a role in the early stage of wound healing after surgery. However, Husain et al. [9]*.* reported a case in which a patient with Kniest dysplasia underwent multiple procedures in the head and neck region, including cochlear implantation, mandibular distraction, palatoplasty, and laryngotracheal reconstruction without any associated complications with respect to wound healing such as soft tissue dehiscence, hypertrophic scar formation, or relapse up to 36 months of age.

Although whether collagenopathy can influence the healing of airway edema or subsequent narrowing of the glottis due to persistent hypertrophy remains unclear, airway assessments are required during the perioperative period, even after growth.

In summary, we have herein presented a patient with Kniest dysplasia in whom trials of tracheal intubation induced laryngeal edema both at the age of 6 years and again at 13 years. Patients with Kniest dysplasia can develop airway narrowing that is not detectable by preoperative interviews or physical examination. Therefore, preoperative assessment of the laryngopharynx and trachea by endoscopy and computed tomography is required to exclude anomalies such as tracheomalacia and airway narrowing. Additionally, because of connective tissue vulnerability, tracheal intubation may also exacerbate airway edema even if the laryngeal condition is normal preoperatively. Therefore, alternative airway management methods such as the use of a laryngeal mask should be considered.
